# Assessment of Genotoxic, Oxidative Stress and Histopathological Effects of Azo Dye Acid Red 119 on Blood and Gill of Freshwater Fish *Channa punctata*

**DOI:** 10.1002/jbt.71092

**Published:** 2026-08-26

**Authors:** Harpal Kaur, Divya Saini, Aditi Khanna, Pooja Chadha

**Affiliations:** ^1^ Department of Zoology Guru Nanak Dev University Amritsar India

**Keywords:** azo dye, *Channa punctata*, genotoxicity, oxidative stress, textile industry

## Abstract

The unregulated release of azo dyes from textile industry effluents into freshwater systems constitutes a serious environmental and toxicological concern. The current investigation was undertaken to elucidate the acute toxicological impact of Acid Red 119 (AR 119) in the blood and gill of freshwater fish *Channa punctata*, using a multi‐tiered biomarker approach encompassing genotoxic, biochemical, and histopathological endpoints. Experimental exposures involved two sub‐lethal concentrations of AR 119−15.90 and 31.81 mg/L‐administered over a 96 h period. The results revealed a dose‐dependent and statistically significant increase in frequencies of micronucleated cells, nuclear and cellular abnormalities, and DNA damage, evidenced by elevated values of comet assay parameters such as tail length and %tail intensity, tail moment, and olive tail moment. Biochemical analysis demonstrated a significant elevation in MDA content, indicating increased lipid peroxidation, along with alterations in SOD and GST activities in blood and gill tissues. The effects were more pronounced at the higher concentration (31.81 mg/L), especially after 96 h of exposure. Histopathological analysis further showed gill tissue damage, including aneurism, epithelial lifting, vacuolization, and fusion of secondary lamellae. Taken together, these findings demonstrated that AR 119 exerts marked genotoxic and oxidative stress in *C. punctata*, underscoring the urgent need for stricter effluent regulation and sustainable treatment strategies in textile industries.

## Graphical abstract

Azo dye Acid Red 119 exerts marked DNA damage, oxidative stress, antioxidant enzymatic, and histopathological alterations in the blood and gills of freshwater fish *Channa punctata*, underscoring the urgent need for stricter effluent regulation and sustainable treatment strategies in textile industries.

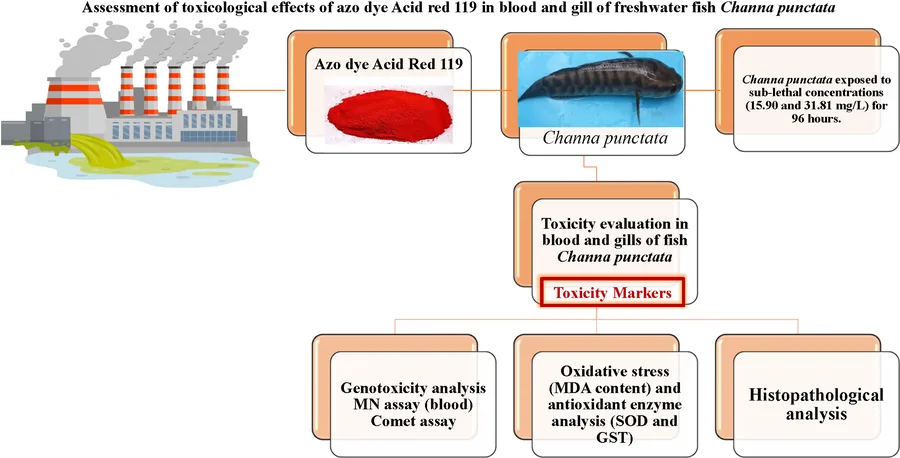

## 1 Introduction

Azo dye pollution in rivers and streams has become one of today's most prevalent environmental issues. Azo dyes are the most significant class of synthetic colorants, widely utilized in the printing, textile, and pharmaceutical industries [1]. They are the aromatic hydrocarbon derivatives of benzene, toluene, naphthalene, phenol, and aniline. They represent a significant class of xenobiotic substances and exhibit resistance to biodegradation processes [2]. Azo dyes are defined by one or more −N=N− (azo bond) and sulfonic (−SO_3_) groups [3]. A significant proportion of azo dye (10%−50% unbound dyes) is vanished in effluent during dyeing procedures [4], causing a big amount of dye to penetrate aquatic bodies [5]. These dyes hinder the photosynthetic ability of aquatic plants as they reduce light penetration and raise biological oxygen demand. As a result, the aquatic organisms are negatively impacted [6, 7].

Azo dyes have the capacity to cause mutagenic, carcinogenic, cytotoxic, and genotoxic effects in a variety of animals because of their electron‐withdrawing nature, impacting the functioning of DNA [8]. Azo dyes can stay in the environment for a very long time because of their half‐life span [2]. Thus, azo dyes are extremely poisonous for aquatic life, particularly in fish, due to their cumulative effect and refractory nature, causing an early reaction by inducing structural and functional changes in many organs. The principal target of these toxicants is thought to be gills [9, 10, 11]. Fish gills make up almost half of their surface area and are the primary organ for respiration, osmoregulation, and excretion [12]. Blood is the most prevalent and significant bodily fluid, and its composition reflects the body's overall physiological state, making it the perfect medium for toxicological investigations [13].

**Figure 1:**
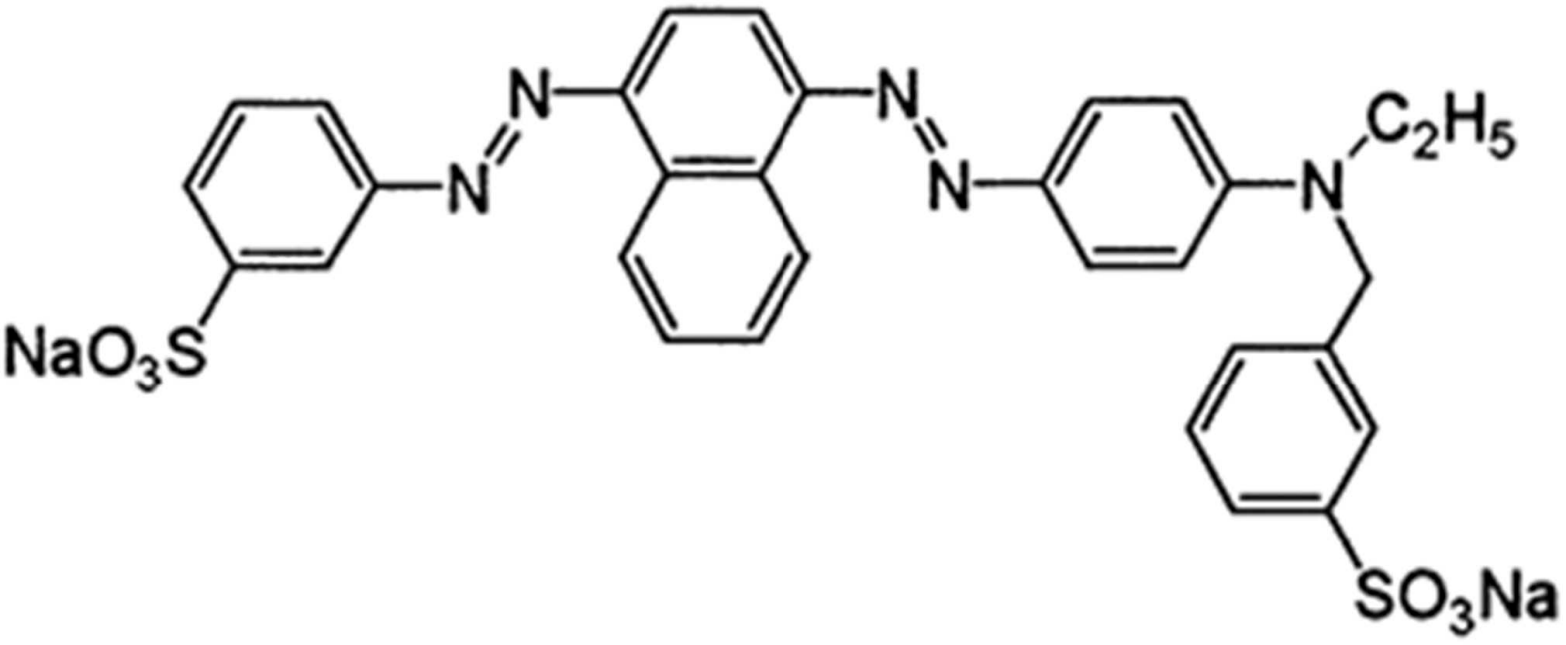
Chemical structure of Acid Red 119.

According to Taqui et al. [14], the azo dye Acid Red 119 (AR 119), frequently detected in textile industry effluents, exhibits potential mutagenic properties. AR 119 is a synthetic red‐colored dye that belongs to the double azo dye class (Figure 1). Acid dyes are mainly used for dyeing silk, wool, nylon, and in printing due to their excellent color fastness [15].

According to the literature review, the toxicity of some azo dyes on aquatic organisms is reported, but the toxic effects induced by AR 119 on aquatic organisms have not been explored to date. Therefore, the current study has been conducted to bridge the knowledge gap in this area. Among various biomarkers used to monitor aquatic toxicity, comet assay is considered to be most promising method for detecting DNA damage because of its sensitivity and speed in evaluating genetic damage [16]. Since histopathology can identify early illness indicators, it is regarded as an important bio‐monitoring tool in toxicity research [17].

In light of these factors, the objective of the study was to assess the toxicological potential of AR 119 in blood and gill of fish *Channa punctata* after acute exposure using genotoxicity, biochemical, and histopathological analysis.

## 2 Materials and Methods

### 2.1 Chemicals

The azo dye AR 119 (molecular formula C_31_H_25_N_5_Na_2_O_6_S_2_; molecular weight, 637.67 g/mol; CAS No.12220‐20‐1; purity: 100%, soluble in water) was procured from Shramik Chemicals, Gujarat, India. The rest of the reagents used in this study were of analytical grade obtained from HiMedia Laboratories Private Limited, Maharashtra, Mumbai, India.

### 2.2 Experimental Organism

Freshwater fish species *C. punctata* (commonly referred to as snakehead fish), with an average body weight of 25 ± 2.11 g and length 12.05 ± 2.31 cm, were obtained from local fish vendors, Amritsar. Upon procurement, the fish were transferred into the containers of approximately 100 L capacity. The fish were then acclimatized to laboratory conditions for a period of 15−20 days to minimize the effects of handling stress and to allow for physiological stabilization before exposure to any experimental procedures. To ensure optimal environmental conditions and prevent the build‐up of nitrogenous wastes, water was replaced on alternate days with dechlorinated tap water. During the acclimation period, the fish were fed daily with a nutritionally balanced synthetic diet (toya pellets).

### 2.3 LC_50_ Determination

Range‐finding experiments for zero percent mortality and 100% mortality were conducted for the determination of LC_50_, and six concentrations were selected for the same. 40 mg/L, 50 mg/L, 60 mg/L, 70 mg/L, 80 mg/L, and 90 mg/L were the finalized doses for the final trials of azo dye AR 119. Fish mortality rates were assessed after 96 h of exposure to varying concentrations of AR 119. Fish were separated into batches of 10 each and subjected to varying doses of AR 119, ranging from 40 mg/L to 90 mg/L. Mortality was monitored at regular intervals of 24, 48, 72, and 96 h following exposure. To ensure the results were reliable, the experiment was conducted three times in duplicates. At 40 and 90 mg/L, the percentage mortality was observed to be 0% and 100%, respectively. The LC_50_ value was calculated by using computer software Probit Analysis [18] and was found to be 63.63 mg/L.

### 2.4 Experimental Design

For the acute exposure experiment, two sub‐lethal doses, ¼ and ½ of LC_50_, were determined. Experiments were carried out in the three groups with each group having six fish. The first group served as a control containing dechlorinated tap water, while the second and third groups were used as exposure groups treated with different concentrations of dye, 15.90 and 31.81 mg/L, respectively. For genotoxic and biochemical analysis, one fish from each group was sampled after 24, 48, 72, and 96 h exposure duration. For histopathological analysis of gill tissue, one fish from each group was sampled after 96 h of exposure. All treatments were conducted in triplicate (*n* = 3).

### 2.5 Water Parameters

Water parameters were analyzed using a water analysis kit. The water quality parameters of water used for acclimatization of fish, control and for the preparation of test solution were as follows: temperature, 26°C–28°C; pH, 7.75–7.78; dissolved oxygen, 6.52–6.55 mg/L; hardness, 220–225 mg/L; total dissolved solids, 306–310 mg/L; conductivity, 515–520 µs/cm; ammonia, < 0.2 mg/L.

### 2.6 Micronucleus (MN) Assay

The MN assay was conducted as outlined by Palhares and Grisolia [19], with minor adjustments. Blood was collected via cardiac puncture using a sterile syringe, and a thin smear was prepared on clean microscopic slides. The smears were air‐dried at room temperature and subsequently fixed in methanol for 10–15 min. Following fixation, the slides were stained with 10% Giemsa solution for 30 min to facilitate visualization of nuclear material. Scoring of micronucleated erythrocytes (MN) and other nuclear and cellular abnormalities was carried out using a trinocular Olympus CX21 microscope equipped with a 3X camera attachment. Initial screening was performed under 10× and 40× magnifications, while detailed evaluation was conducted under 100× using immersion oil. For each specimen, a total of 1000 erythrocytes were examined in both control and treatment groups to assess genotoxic effects.

### 2.7 Comet Assay

To assess DNA damage in blood and gill, the comet assay was carried out based on the methodology of Singh et al. [20]. DNA migration patterns (comet formation) were visualized using a fluorescence microscope equipped with a 40× magnification objective. Quantitative analysis of DNA damage was performed using CASP software, evaluating parameters such as tail length (TL), percentage tail intensity (%TI), tail moment (TM), and olive tail moment (OTM).

### 2.8 Biochemical Analysis

In order to investigate acute toxicity, blood and gill samples were taken at 24, 48, 72, and 96 h intervals. Blood was drawn directly from the heart on each sampling day. The samples were subsequently centrifuged for 10 min at 3000 rpm. The obtained supernatant was diluted in a 1:10 (v/v) ratio with PBS (0.1 M, pH 7.4). Samples were then promptly kept for later analysis at −20°C.

The gill tissues were removed from each group and cleaned with phosphate buffer after 24, 48, 72, and 96 h of exposure. Using a Remi BZ‐004 tissue homogenizer, 10% homogenate (1:10 w/v ratio) was made in phosphate buffer saline (0.1 M, pH 7.4). The homogenate was centrifuged in a Remi centrifuge C‐24 PLUS for 10 min at 10,000 rpm at 4°C. After centrifugation, the supernatant was extracted and immediately stored in aliquots at −20°C for further examination. For analysis, the Eppendorf Biospectrometer Kinetic was employed. The measurement of lipid peroxidation followed the methodology of Draper and Hadley [21], while SOD and GST activities were determined using the methods described by Kono [22] and Habig and Jakoby [23], respectively.

### 2.9 Histopathological Analysis

At the end of 96 h exposure, gill tissue samples from each group were collected and fixed in 10% buffered formalin for 24 h. Excess fixative was removed by rinsing the samples in running water. The tissues were then dehydrated in increasing concentrations of alcohol, cleared in xylene, and embedded in paraffin wax. Staining was carried out using hematoxylin and eosin, and microscopic examination was conducted to assess histological abnormalities in the gills.

### 2.10 Statistical Analysis

Data analysis was done using SPSS software (version 21.0). Results were expressed as mean ± standard error (SE). The study design ensured that observations were independent. Normal distribution and homogeneity of variance were assessed using the Shapiro–Wilk and Levene's tests, respectively. Since the data met the criteria for one‐way ANOVA, therefore one‐way ANOVA with Tukey's post hoc comparison was performed, with significance determined at *p* ≤ 0.05. Further, the data were reanalyzed using two‐way ANOVA with concentration and exposure duration as independent factors (Supporting Information S1: Tables 1 and 2).

### 2.11 Ethical Statement

All experimental protocols were approved by the Institutional Animal Ethics Committee (IAEC) of Guru Nanak Dev University, Amritsar (Approval No. 226/CCSEA/2024/02). The study was conducted in full compliance with the ethical standards and regulations set forth by the Committee for the Purpose of Control and Supervision of Experiments on Animals (CCSEA), Government of India.

## 3 Results

### 3.1 Genotoxic Effect

The genotoxic effect of azo dye AR 119 was evaluated using the MN assay and comet assay in blood and gill cells after 24, 48, 72, and 96 h of exposure.

#### 3.1.1 MN Assay

**Figure 2:**
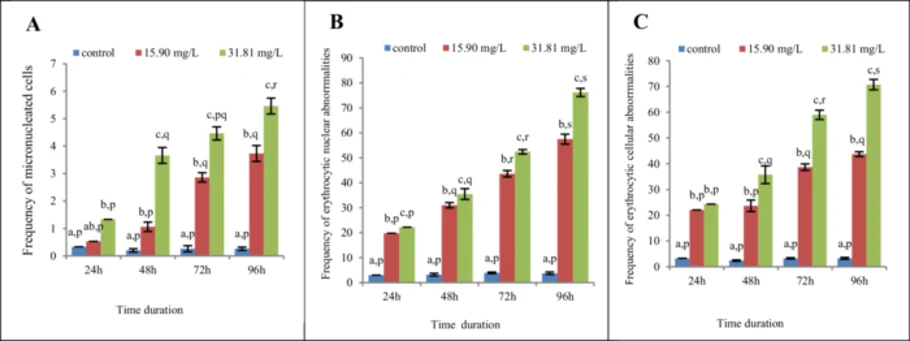
Influence of varying concentrations of AR 119 on frequency of (A) micronucleated cell, (B) erythrocytic nuclear abnormalities (ENA), and (C) erythrocytic cellular abnormalities (ECA) in blood cells of *Channa punctata* at different durations of exposure. Error bars represent standard errors (SE). Different letters p, q, r, s signify the effect of durations of exposure, and a, b, c signify the effect of treatment at the same time interval. One‐way ANOVA followed by Tukey's post hoc test was performed using SPSS software (version 21.0).

**Figure 3:**
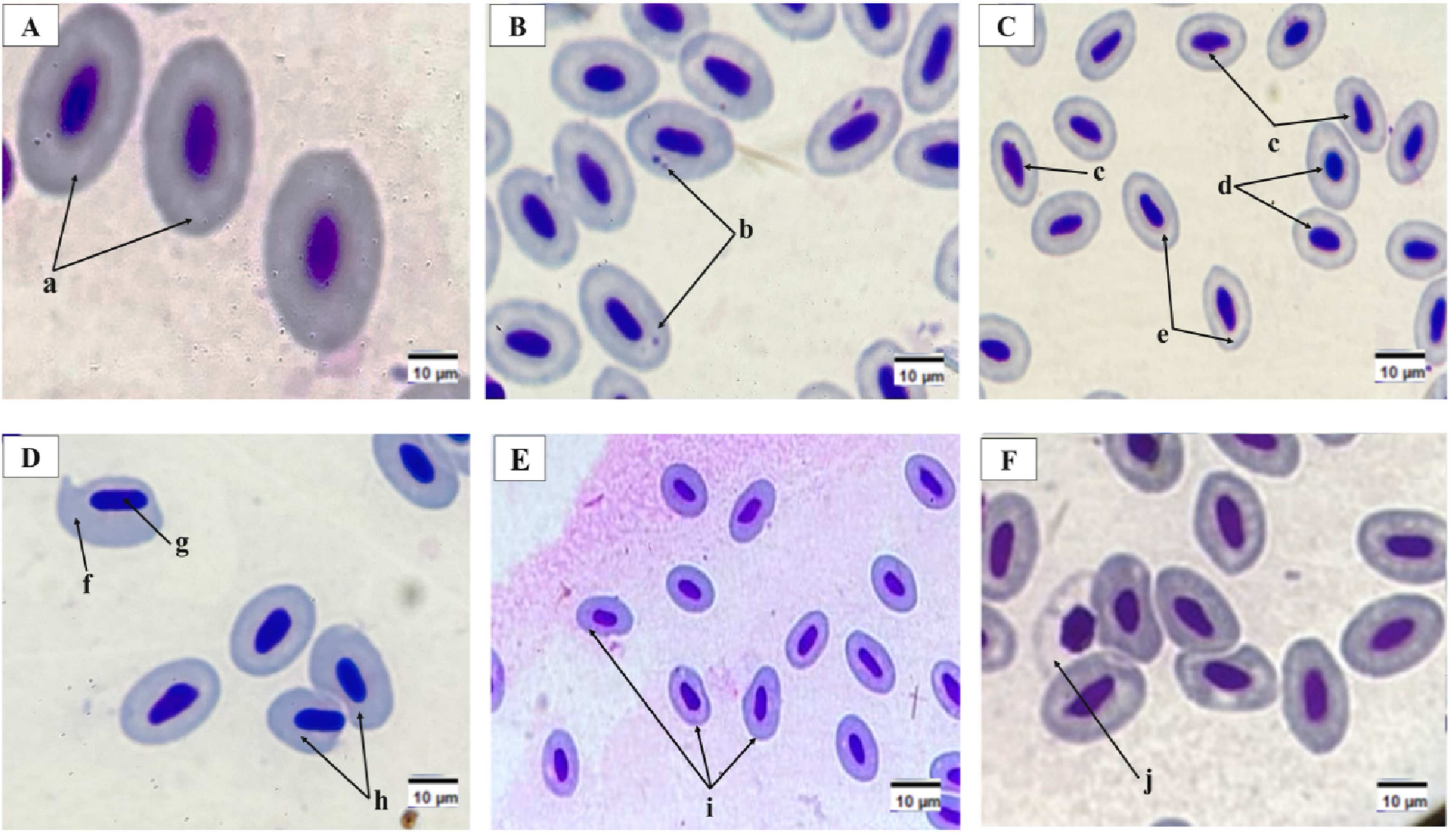
Photomicrographs presenting different anomalies in blood cells of *Channa punctata* after exposure to AR 119 (A) control, (B−D) 31.81 mg/L exposed group, (E, F) 15.90 mg/L exposed group. (a) normal cells, (b) micronucleated cells, (c) notched nucleus, (d) round‐shaped nucleus, (e) vacuolated cytoplasm, (f) hook‐shaped cell, (g) elongated nucleus, (h) fragmented cells, (i) irregular‐shaped cells, (j) lysed cell. Image at 100× objective lens, scale bar‐10 µm.

The present study reveals the frequency of micronucleated cells (MN), erythrocytic nuclear abnormalities (ENA), and erythrocytic cellular abnormalities (ECA) in blood cells of fish *C. punctata* after exposure to azo dye AR 119 for different time intervals (Figure 2). MN, ENA, and ECA frequency was obtained to be significantly (*p* ≤ 0.05) higher in 15.90 and 31.81 mg/L exposed groups relative to the control group. The highest value of MN frequency was observed after 96 h of exposure in the 31.81 mg/L exposed group. The value was found to be 4.03‐fold, 18.30‐fold, 17.15‐fold, and 21.00‐fold increased as compared to control after 24, 48, 72, and 96 h of duration, respectively. A 20.41‐fold and 21.69‐fold rise in ENA and ECA frequency was observed after 96 h of exposure. Different nuclear and cellular abnormalities were observed in blood cells such as micronucleated cells, hook‐shaped cell, fragmented cells, irregular shaped cells, notched nucleus, round‐shaped nucleus, vacuolated cytoplasm, elongated nucleus, and lysed cells (Figure 3).

#### 3.1.2 Comet Assay

**Figure 4:**
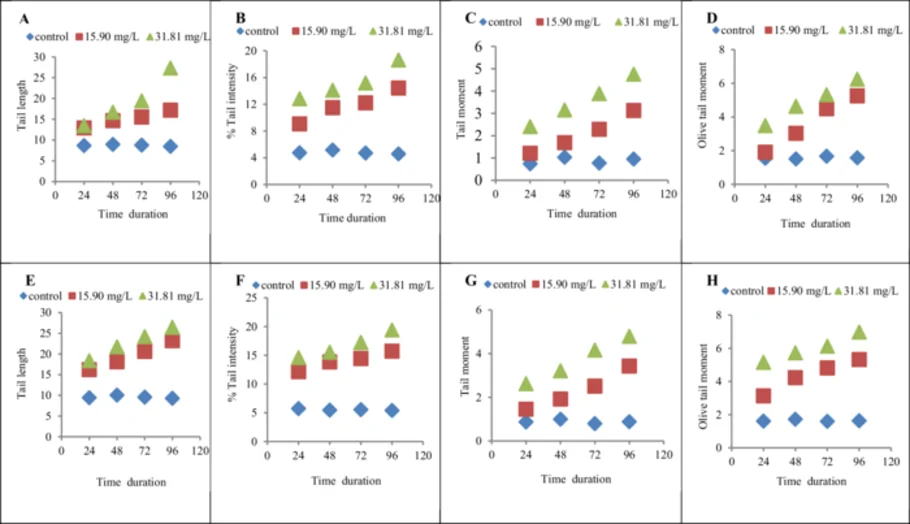
Scatter plots showing the alterations in comet parameters (A−D) in blood cells and gill cells (E−H) of *Channa punctata* exposed to varying concentrations of azo dye AR 119 at different durations of exposure.

**Figure 5:**
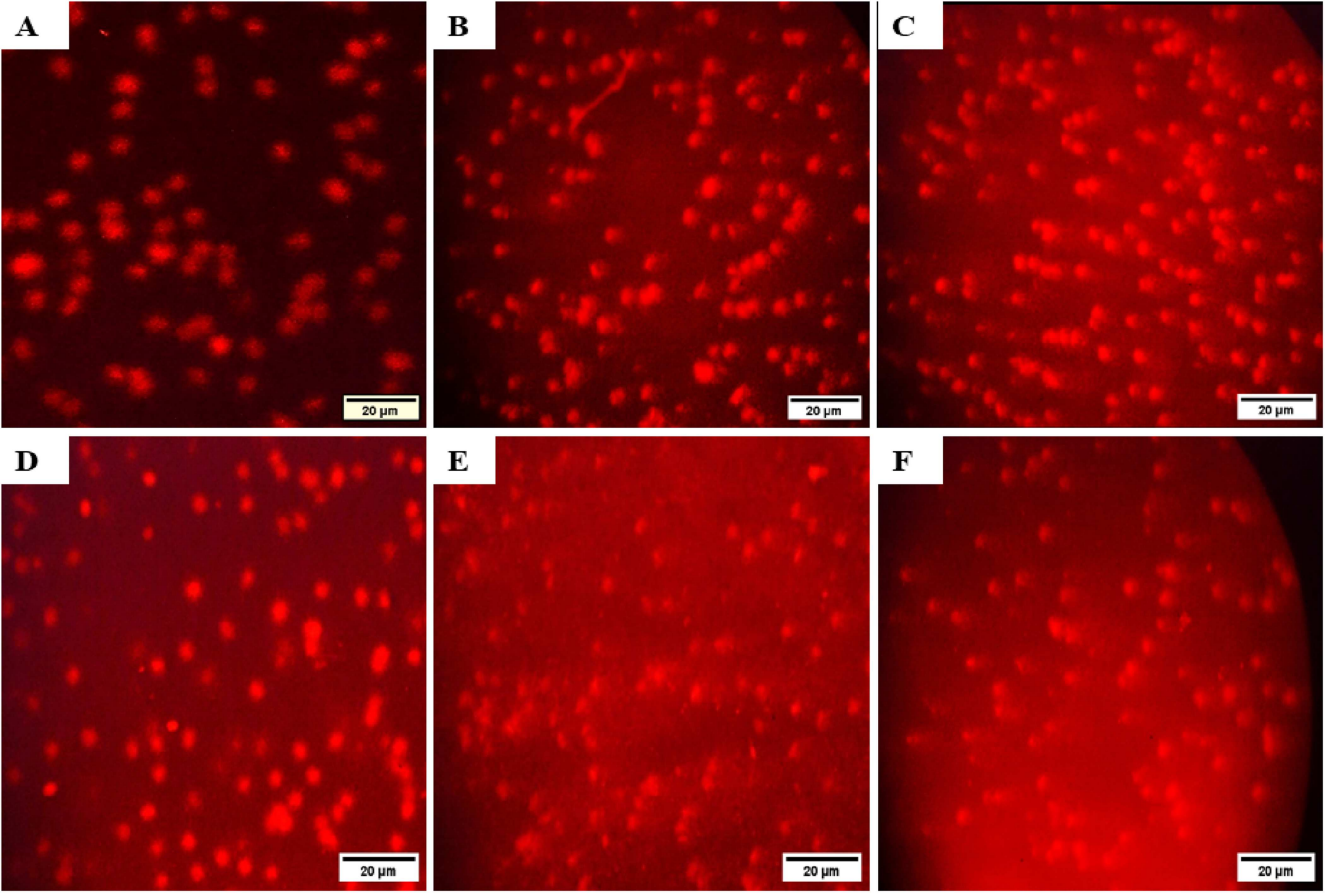
Photomicrographs showing different DNA damage in blood and gill cells of *Channa punctata* after exposure to AR 119 (A) control blood cells, (B) 15.90 mg/L exposed group, (C) 31.81 mg/L exposed group, (D) control gill cells, (E) 15.90 mg/L exposed group, (F) 31.81 mg/L exposed group. Ethidium bromide‐stained images at 40× objective lens, scale bar‐20 µm.

Figure 4 illustrates the extent of DNA damage in the blood and gill of *C. punctata* following exposure to two concentrations of AR 119 (15.90 and 31.81 mg/L) across various time intervals. Photomicrographs showing DNA damage in blood and gill tissues of *C. punctata* after being exposed to AR 119 are represented in Figure 5. The values of TL, %TI, TM, and OTM were found to be significantly higher (*p* ≤ 0.05) in both blood and gill tissues of the 15.90 mg/L concentration exposed group after 96 h of exposure as compared to the control group. Maximum damage was observed in 31.81 mg/L concentration exposed blood and gill tissues after 96 h of exposure. In case of blood, TL increased from 8.66 ± 0.12 to 26.41 ± 0.55 µm, %TI increased from 4.58 ± 0.09 to 18.16 ± 0.25, TM increased from 0.93 ± 0.05 to 4.86 ± 0.05, and OTM increased from 1.67 ± 0.02 to 7.07 ± 0.05 after 96 h of exposure in the 31.81 mg/L exposed group as compared to the control group, respectively. However, 2.78‐fold, 3.50‐fold, 5.10‐fold, and 4.23‐fold rise in TL, %TI, TM, and OTM were observed in gill tissue after 96 h of exposure.

### 3.2 Biochemical Analysis

Acute exposure to AR 119 induced a marked change in the antioxidant defense system of *C. punctata*. In blood and gill tissues, biochemical analysis was done in which different biochemical markers like malondialdehyde (MDA) content, SOD, and GST activities were studied. The results for all the biomarkers have been summarized below.

#### 3.2.1 MDA Level

**Figure 6:**
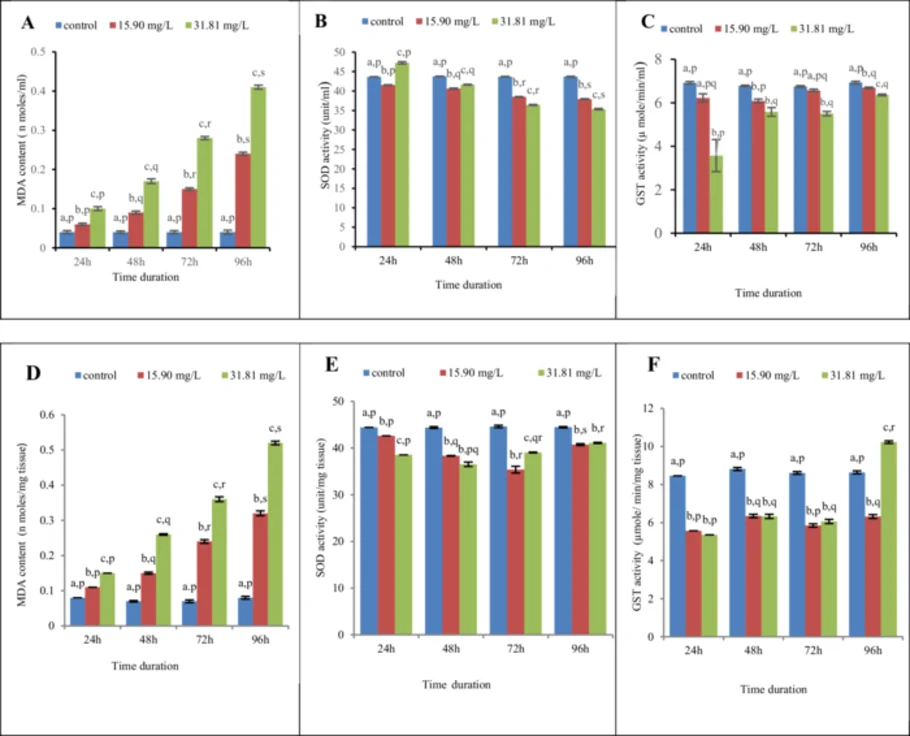
Impact of varying concentrations of AR 119 on MDA content, SOD activity, and GST activity in blood (A−C) and gill (D−E) of *Channa punctata* at different durations of exposure. Error bars represent standard errors (SE). Different letters p, q, r, s signify the effect of durations of exposure, and a, b, c signify the effect of treatment at the same time interval. One‐way ANOVA followed by Tukey's post hoc test was performed using SPSS software (version 21.0).

A significant rise in the MDA level was observed in both 15.90 and 31.81 mg/L concentration exposed groups at all durations as compared to the control group (ANOVA) (Figure 6). In blood tissue, the maximum effect was discerned after 96 h of duration to 31.81 mg/L concentration of dye. Value was found to be 10.25‐fold increase relative to the control group after 96 h duration. However, in the gill tissue, a 1.37‐fold and 1.87‐fold rise in 15.90 and 31.81 mg/L concentrations was noticed in contrast to the control group after 24 h of exposure. Further, with the increase in the duration of exposure, a significant hike in MDA content was noticed. The highest effect was observed after 96 h of duration to 31.81 mg/L concentration of AR 119, and the increase was 6.50‐fold as compared to the control after 96 h of exposure.

#### 3.2.2 SOD Activity

Figure 6 reveals a significant reduction in SOD activity in blood of *C. punctata* in both the exposed groups, that is, 15.90 and 31.81 mg/L, as compared to the control group. After 24 h of exposure, SOD activity was decreased to 41.54 ± 0.06 (15.90 mg/L) from 43.65 ± 0.03 (control). Further, with an increase in exposure duration as well as an increase in concentration, in comparison to the control group, SOD activity was found to be decreased. A reduction of 13.25% in activity of SOD was observed after 96 h of exposure. However, the 31.81 mg/L concentration exposed group showed an increase in SOD activity (47.25 ± 0.30) in comparison to the control group (43.65 ± 0.03) after 24 h of exposure. After that, significant decline in SOD activity was noted after 48 h followed by 72 and 96 h of exposure.

In 15.90 mg/L concentration exposed gill tissue, a gradual decrease in SOD activity was recorded after 24 h of exposure, followed by 48 and 72 h. Maximum 20.70% reduction was noted at 72 h of exposure as compared to the control group. Further, SOD activity observed to be increased from 72 h of exposure to 96 h of exposure (35.39 ± 0.19 to 40.76 ± 0.23), but overall activity of SOD perceived to be decreased as compared to the control group. In 31.81 mg/L concentration exposed group, maximum 17.72% reduction was noticed at 48 h of exposure as compared to control group. Further, a rise in SOD activity after 48 h of exposure was noted, but overall activity of SOD was observed to have declined as compared to the control group.

#### 3.2.3 GST Activity

After acute exposure, there was a significant decline in GST activity in blood tissue of both 15.90 and 31.81 mg/L exposed groups in contrast with the control group (Figure 6). Comparing the 15.90 mg/L concentration exposed group to control group, 10.11%, 10.32%, 2.66%, and 3.46% reduction was observed after 24, 48, 72, and 96 h of exposure, respectively. Similarly, on comparing the GST activity of 31.81 mg/L concentration exposed group with the control group, 48.41%, 17.69%, 18.51%, and 8.22% reduction were detected after 24, 48, 72, and 96 h of exposure, respectively.

Altered activity of GST in gill tissue of *C. punctata* after exposure to two concentrations (15.90 and 31.81 mg/L) of AR 119 are depicted in Figure 6. After 24 h of exposure, a decrease in GST activity was noted in both the exposed groups. 34.16% and 36.64% reduction was noted in 15.90 and 31.81 mg/L concentration exposed groups as compared to the control group. However, in higher concentration (31.81 mg/L) after 96 h of exposure, a 1.18‐fold rise in GST activity was recorded as compared to control group.

### 3.3 Histopathological Analysis

**Figure 7:**
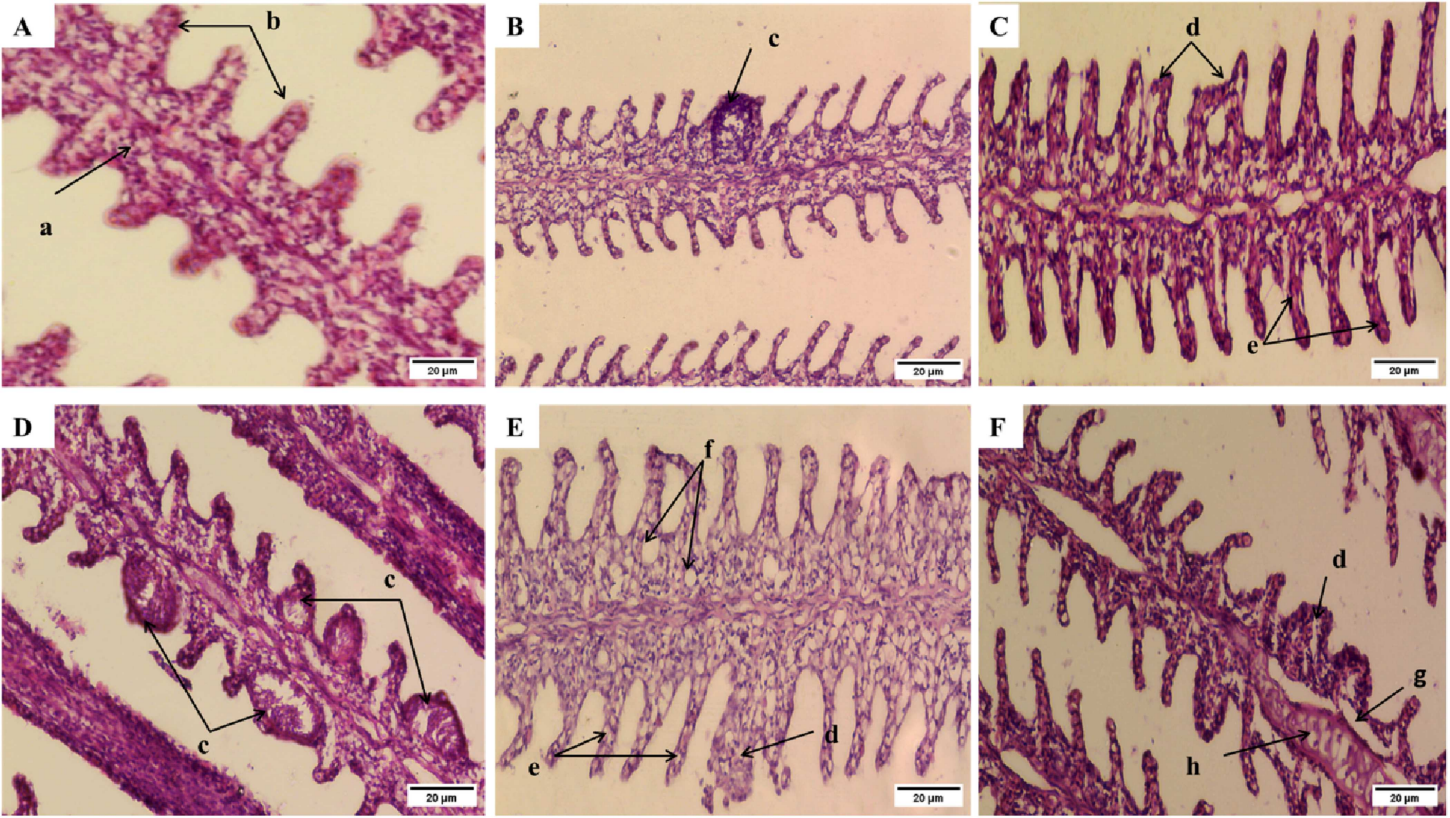
Illustrates the histopathological effect of Acid Red 119 in gill tissue of *Channa punctata*. (A) control group, (B, C) 15.90 mg/L concentration exposed group, (D–F) 31.81 mg/L concentration exposed group. (a) Normal primary lamellae, (b) normal secondary lamellae, (c) aneurism, (d) fused secondary lamellae, (e) elongated secondary lamellae, (f) vacuolization, (g) upliftment of gill epithelial layer, (h) swelling of blood vessels. Hematoxylin and eosin‐stained images at 40× objective lens, scale bar‐20 µm.

The gills of the control group fish showed well‐defined primary and secondary gill lamellae with no significant lesions. Compared with the control group, the gills of exposed fish exhibited considerable damage. AR 119 treatment at concentrations of 15.90 and 31.81 mg/L for 96 h caused a variety of detrimental alterations in the architecture of the gills. Alterations such as fusion and elongation of secondary lamellae, aneurism were recorded in 15.90 mg/L concentration, whereas numerous anomalies were detected at a higher 31.81 mg/L concentration included multiple aneurism, vacuolization, fusion and elongation of secondary lamellae, upliftment of gill epithelial layer, and enlargement of blood vessels (Figure 7). The incidence and severity scores for gill tissue were found to be higher in the dye‐exposed groups as compared to the control group (Supporting Information S1: Table 3).

## 4 Discussion

The current study was conducted to assess the toxic effects of the azo dye AR 119, using parameters like genotoxicity (MN assay and comet assay), biochemical and histopathological analysis in blood and gill tissues of the fish *C. punctata*.

The genotoxic potential of AR 119 was assessed using the MN assay and comet assay in the blood and gill of *C. punctata*. These assays are widely recognized for their simplicity, sensitivity, and ability to detect DNA damage at the cellular level [24]. These techniques collectively offer a comprehensive evaluation of genotoxic stress, enabling the detection of DNA damage induced by environmental toxicants.

Since most of the textile azo dyes are hydrophilic, they can easily enter the fish body from their surroundings, either by absorption through skin and gills or by ingestion, leading to their accumulation in target tissues. Because of their recalcitrant properties, these dyes can damage the concerned tissue and have a negative impact on fish physiology [25]. The majority of azo dyes undergo reduction catalyzed by enzymes of the intestinal microorganisms and hepatic enzymes that cleave the ─N═N─ bond, generating sulfonated aromatic amines. Most of the water‐soluble dyes become mutagenic only after reduction. AR 119 is a diazo dye contains azo (─N═N─) bonds, which are susceptible to enzymatic reduction, leading to the formation of sulfonated aromatic amines. These amine metabolites are known to generate reactive oxygen species (ROS), potentially resulting in oxidative stress, lipid peroxidation, and disruption of mitochondrial function [15, 26].

In the existing study, a higher frequency of micronucleated cells, nuclear and cellular abnormalities were observed in both concentrations, that is, 15.90 and 31.81 mg/L after acute exposure to AR 119 as compared to control group. Moreover, comet parameters such as TL, %TI, TM, and OTM were assessed to evaluate the DNA damage. A significant elevation in all the comet parameters were detected after exposure to both 15.90 and 31.81 mg/L concentrations relative to control group, and maximum damage was observed in 31.81 mg/L concentration‐exposed group after 96 h of exposure.

The genotoxic effects observed may result from the interaction of toxicants with DNA, causing strand breaks, crosslinks, and adduct formation, which impair transcription and cellular function [27, 28]. Nuclear aberrations such as vacuolated cytoplasm, buds, and elongated nuclei likely arise from chromosomal missegregation and gene amplification induced by pollutants, leading to distorted nuclear forms [29, 30]. Likewise, the increase in the comet assay parameters observed in the present study may be attributed to elevated free radical generation, which interacts with DNA and other biomolecules, leading to the formation of DNA adducts and subsequent DNA damage [31, 32]. Kirandeep et al. [33] studied the cytogenotoxicity of Acid Blue‐113 on *Channa punctatus* and found that with an increase in concentration and duration, frequency of MN, cytoplasmic, and nuclear abnormalities were also intensified. Cytogenotoxicity of Reactive Red 120 (RR120) was assessed in gills and blood cells of freshwater fish *Catla catla* by Parmar and Shah [34]. Dose and time‐dependent increase in micronuclei, fragmented apoptotic and bi‐nucleated cells, and nuclear buds were observed in both peripheral blood and gill cells.

Many aerobic cellular metabolic processes are responsible for production of ROS [35]. Oxidative stress can induce destruction to DNA, lipids, and proteins primarily through the excessive production of ROS, leading to the loss of structural and functional integrity of enzymes and the activation of inflammatory responses [36]. Lipid peroxidation, resulting from the reaction of oxygen with unsaturated lipids, generates a diverse range of oxidation products with MDA being one of the most mutagenic products [37]. In the current study, concentration and duration‐dependent increase in MDA content was observed in the blood and gill tissues of fish. The observed increase in MDA levels is probably accredited to excessive production of ROS [38]. Elevated ROS levels have been reported to induce cellular damage when their rate of generation exceeds the capacity of antioxidant defense mechanisms to neutralize them [39].

SOD assists to guard cells against the oxidative damage of free radicals by conversion of superoxide radical into hydrogen peroxide and molecular oxygen species [35]. In the present study, in comparison to the control group, there was a substantial decrease in SOD activity in blood and gill tissues of both the concentration‐exposed (15.90 and 31.81 mg/L) groups after 96 h of exposure. The observed decline in SOD activity can be linked to the accumulation and overproduction of superoxide anion radicals [40, 41].

GST is a prominent Phase II detoxification enzyme that facilitates the binding of glutathione to a range of xenobiotics and their metabolic by‐products [42]. In fish, GST plays an essential role in detoxifying and eliminating environmental contaminants [43]. In the current study, a reduction in GST activity was observed in the blood cells of *C. punctata* after 96 h of exposure to both 15.90 and 31.81 mg/L concentrations, compared to the control group. But in gill tissue, GST activity was reported to be hiked after 96 h of exposure in 31.81 mg/L concentration exposed group as compared to control group. The increase in GST activity might be associated with defense against oxidative damage caused by the dye [2, 44]. Kaur and Kaur [2] demonstrated a decrease in the activity of SOD and an increase in the activity of GST of *Labeo rohita* exposed to azo dye, Acid black. Consistently, Ayadi et al. [45] also reported a decrease in GST activity in the liver and gill tissues of *Oreochromis niloticus* exposed to varying concentrations of Red 195 dye. The study presented by Fathima et al. [46] also showed an increase in lipid peroxidation in the gills of acid orange 7‐exposed *Anabas testudineus*. As observed in the present study, a significant increase in MDA and a decrease in SOD activity had also been noticed in *Carassius carassius* exposed to tartrazine [47]. On exposure to the azo dye Sunset Yellow, there was a diminution in GST activity in zebrafish larvae, indicating the induction of oxidative stress [48].

By using histopathological methodology, it becomes simple to spot structural alterations in various organs when compared to their functional counterparts. It may also act as an early warning system for animal health issues [17, 49]. The benefit of employing histopathological techniques for environmental monitoring is that it enables us to look at particular organs, such as the kidney, liver, and gills. Since gills readily respond to xenobiotic exposure and are very vulnerable to contaminants even at very low concentrations, they are suitable organs for histological examination to determine the effects of dye. Thus, in the current study, gills are taken into consideration for histopathology assessment in order to assess aquatic contamination in fish because of their direct interaction with contaminants in the water [50]. The control group showed no alterations in the histology of gill tissue in the present study, but dye‐exposed groups (15.90 and 31.91 mg/L) showed severe alterations in histology such as vacuolization, aneurism, fusion of secondary lamellae, upliftment of gill epithelial layer, elongation of secondary lamellae, and enlargement of blood vessels. Secondary lamellae fusion is a defense strategy because it reduces the amount of exposed gill surface and creates a barrier between blood and pollutants [51]. The penetration of contaminants into the basement membrane and epithelium may be the cause of the upliftment of the gill epithelial layer, which ultimately increases the diffusion distance for gaseous exchange [52]. Blood vessel enlargement causes neighboring cells to proliferate, reducing the interlamellar space and ultimately causing lamellar fusion [53]. It reduces the surface area available for gas exchange. Due to the severance of pillar cells, the aneurysm is considered to be an accumulation of blood in the secondary lamellae, resulting in increased blood flow that promotes bleeding. The gills’ blood transport mechanism is also hampered [54, 55]. The present study is corroborated by the work of various researchers. Jagruti [56] studies several alterations in the histology of fish *C. catla* exposed to dye reactive red 120. Histopathological alterations such as hyperplasia, degenerated central axis, aneurism, curved secondary lamellae, and lifted gill epithelium were observed in gill tissues. Similarly, Kaur et al. [57] found epithelial lifting, lamellar fusion, increased gill filament, necrosis, and mucus deposition in gills of fish *L. rohita* exposed to azo dye basic violet 1. Study reported by Amwele et al. [58] investigated the impact of dark green azo dye; Anionic surfactant oil or mixture of these two substances on gills in fish *O. niloticus*. Epithelial hypertrophy, secondary lamellae hyperplasia, primary lamellae lifting, lamellae aneurysm, edema, and blood congestion were observed in gill pathologies. Parmar and Shah [59] also recorded the toxicity of azo dye acid red 97 using histopathology in fish *C. catla*. Swelling, aneurysm, lifted epithelial layer, shorter and severe erosions of secondary lamellae were noticed in gill tissue exposed to two sublethal concentrations 0.85 and 1.7 mg/L for 10, 20, and 30 days.

### 4.1 Limitations of the Study

The present study provides clear evidence of genotoxicity, oxidative stress, and histopathological alterations induced by AR 119 in the freshwater fish *C. punctata*; however, some limitations should be acknowledged. First, the investigation was restricted to acute exposure (96 h) under controlled laboratory conditions and may not fully represent long‐term environmental exposure scenarios. Second, while biomarker responses were assessed at the cellular, biochemical, and tissue levels, the precise molecular mechanisms responsible for the observed toxic effects were not directly investigated.

### 4.2 Future Research Directions

Further research focusing on chronic exposure studies using environmentally relevant concentrations of AR 119 will be carried out in the future. Investigations into the bioaccumulation and trophic transfer of the dye, as well as its effects on growth, reproduction, and survival, would improve ecological risk assessment. Molecular studies such as gene expression changes related to antioxidant defense, apoptosis, and DNA repair would further clarify the mechanisms underlying toxicity. Additionally, studies involving mixtures of textile dyes and other environmental contaminants would provide a more realistic assessment of risks in freshwater ecosystems.

## 5 Conclusion

The outcomes of the existing study clearly demonstrated that AR 119 is capable of inducing genotoxicity and oxidative stress in the blood and gill tissues of *C. punctata* following acute exposure. Study further revealed the histopathological anomalies in gill tissue of *C. punctata*. This study provides valuable toxicological insights into the effects of AR 119 on fish *C. punctata*. Further, toxicological evaluations incorporating a broader range of biomarkers are necessary to comprehensively assess its environmental impact on aquatic organisms. The study underscores the urgent need for more stringent regulation of azo dye discharge into aquatic environments, along with the implementation of advanced, eco‐friendly treatment technologies in textile wastewater management. Addressing these concerns is imperative to safeguard aquatic biodiversity and mitigate long‐term ecological risks associated with synthetic dye pollution.

## Author Contributions

**Harpal Kaur:** conceptualization, writing – original draft, data curation, investigation. **Divya Saini:** investigation, data curation. **Aditi Khanna:** investigation, data curation. **Pooja Chadha:** supervision, writing – review and editing.

## Conflicts of Interest

The authors declare no conflicts of interest.

## Supporting information


Supporting File

## Data Availability

The data that support the findings of this study are available from the corresponding author upon reasonable request.
