# Supplementary material for: Assessment of Genotoxic, Oxidative Stress and Histopathological Effects of Azo Dye Acid Red 119 on Blood and Gill of Freshwater Fish Channa punctata
Source: J Biochem Mol Toxicol. 2026 Aug 26;40(9):e71092. doi: 10.1002/jbt.71092 (PMC13511308; doi:10.1002/jbt.71092)
Supplement: Supplementary file 1 [file JBT-40-e71092-s001.docx]

**Table 1. Two-way ANOVA for the effects of AR 119 concentrations and duration (exposure time) on blood parameters measured in *Channa punctata*.**

| **Parameters** | **Concentration** | **Duration** | **Concentration x Duration** | **Partial eta squared** |
| --- | --- | --- | --- | --- |
| MN | 279** | 78** | 23** | 0.854 |
| ENA | 742** | 188** | 49** | 0.925 |
| ECA | 761** | 128** | 41** | 0.913 |
| TL | 2452** | 372** | 161** | 0.976 |
| %TI | 4511** | 262** | 69** | 0.946 |
| TM | 2407** | 431** | 108** | 0.964 |
| OTM | 2187** | 504** | 124** | 0.969 |
| MDA | 1807** | 644** | 191** | 0.980 |
| SOD | 1097** | 960** | 443** | 0.991 |
| GST | 48** | 11** | 7** | 0.667 |

Data were expressed by F value, ** represent p≤ 0.001 (95% confidence intervals).

**Table 2. Two-way ANOVA for the effects of AR 119 concentrations and duration (exposure time) on gill parameters measured in *Channa punctata.***

| **Parameters** | **Concentration** | **Duration** | **Concentration x Duration** | **Partial eta squared** |
| --- | --- | --- | --- | --- |
| TL | 4377** | 316** | 82** | 0.954 |
| %TI | 6044** | 201** | 55** | 0.933 |
| TM | 2407** | 431** | 106** | 0.945 |
| OTM | 2542** | 134** | 34** | 0.896 |
| MDA | 755** | 494** | 546** | 0.993 |
| SOD | 261** | 34** | 27** | 0.871 |
| GST | 741** | 223** | 153** | 0.975 |

Data were expressed by F value, ** represents p≤0.001 (95% confidence intervals).

**Table 3**

The table showing the data on the incidence and severity score for gill tissue in different treatment groups.

| Name of the organ | Name of the alterations in gill tissue | Treatment groups | | | | | |
| --- | --- | --- | --- | --- | --- | --- | --- |
|  |  | Control group | | 15.90 mg/L concentration | | 31.81 mg/L concentration | |
| Gill tissue |  | Incidence | Severity | Incidence | Severity | Incidence | Severity |
|  | Fusion and elongation of secondary lamellae | 1 | I | 3 | II | 4 | II |
|  | Aneurism | 0 | 0 | 2 | II | 4 | II |
|  | Vacuolization | 1 | I | 2 | II | 4 | II |
|  | Upliftment of gill epithelial layer | 0 | 0 | 1 | I | 3 | II |
|  | Enlargement of blood vessels | 0 | 0 | 0 | 0 | 4 | II |

Incidence: - The number of times for any alteration observed. For example: 1, 2, 3… etc.

Severity Grade: - Stage I (alterations that do not affect tissue functioning), Stage II (severe damage, obstructing the normal functioning of the tissue) and Stage III (extremely high damage).
